# Periscope of Dermatology

**Published:** 1899-09-09

**Authors:** 


### PERISCOPE OF DERMATOLOGY.

(*Continued from page* 386.)

Deas[10] records the case of a man, aged 34, who was suffering from herpes, affecting the skin supplied by the eleventh and twelfth dorsal, and the first lumbar nerves. Antipyrin, in 10 grain doses, was given every four hours, and about a quarter of an hour after the first dose an erythematous rash developed all over the body, which was succeeded by bullæ; these, however, disappeared within eight hours. The skin affected by herpes was unaffected by the drug rash. There was no reappearance of the rash, although the patient continued the drug.

**Rontgen Rays.**—Albers-Schönberg[11] has employed the Röntgen rays in the statement of lupus, chronic eczema, favus, and psoriasis. A considerable time must elapse before any positive treatment can be relied on with regard to their healing power. In a case of lupus, after the first five or six exposures the affected part becomes red, and the temperature of the part becomes raised. Later the tubercles become enlarged, scabs form, which soon separate, leaving a clean ulcer. The infiltration of surrounding parts disappears, and the skin becomes smooth and free from all lupoid tissue. No advantage is gained by increasing the number or length of the exposures, since the process of repair would be much delayed thereby. There does not appear to be less likelihood of recurrence than after milder methods of treatment. The treatment of chronic eczema has been very successful, the skin being left with some pigmentation, but otherwise normal. Obstinate cases were selected in which all other treatment had failed. The action of the rays consists in drying up the skin, and then causing it to desquamate. He also successfully treated a case of favus, without any destruction of the hair.

Galloway[12] refers to two reports of cases of legal action being taken against physicians on account of sores produced by the X rays. In one case the rays were used for diagnostic and in the other for therapeutic purposes; in this latter case a circular callous sore resulted. He urges caution in the employment of the rays, especially in neurotic women.

**Various: Mycosis Fungoides.**—McNeil, Murray, and Atkinson[13] report a case of mycosis fungoides, which occurred on the head of a man aged 43, and which apparently resulted from a kick from a goat. The whole of the face and scalp was covered by rapidlygrowing soft tumours; from time to time there were febrile attacks, accompanied by a sort of erysipelatous eruption on the face, during the existence of which the tumours seemed to decrease in size. A bacteriological examination was made, and a pure cultivation of a special bacillus was obtained; this organism was pathognomic to rabbits, since it produced swellings at the points of inoculation, and, after inoculation into the peritoneal cavity, death after a few hours. This is the first time that a definite micro-organism has been isolated in this disease.

**Desquamative Dermatitis.**—Crocker[14] records two cases of recurrent desquamative scarletiniform dermatitis. This disease is characterised by the rapid and universal invasion of the whole body with an erythematous efflorescence, followed by desquamation in large flakes; there is also a marked tendency to relapse and, sooner or later, to recur at regular intervals of months or years. In the absence of previous history the important points in diagnosis are the short duration of the podromal symptoms, the early occurrence of desquamation (within three or four days), and the absence of any elevation of temperature. Warmth is the only treatment required, but salicin internally has prevented a relapse.

**Pediculosis.**—Jamieson[15] states that the cause of the reappearance of pediculi vestimentorii after apparently thorough treatment is to be found in the fact that the eggs of the pediculi may be discovered on some of the short hairs of the body, especially on the back between the shoulders. He therefore recommends that paraffin should be rubbed in all over the body, and that the patients should then be given a warm carbolic bath. He suggests that the term pediculi corporis is preferable to pediculi vestimentorii.

**Lichen Annularis.**—Galloway[16] records a case of lichen annularis in which a peculiar eruption occurred on the hands. The patient was a boy aged ten, and the affection had existed for three years. Each lesion consisted of a pale ivory-like elevated border showing a circular or circinate outline in the neighbourhood of the dorsal and lateral aspects of the joints of the hand and fingers. Portions of one lesion were removed for microscopic examination, but only cell infiltration was found, no micro-organisms being detected. The case improved after the application of a 10 per cent. salicylic acid ointment. Lindley Scott[17] reports five cases of skin eruption occurring during the course of nephritis. Of the five cases, four were males and one was a female; the ages varied from 16 to 54 years. In its early stage the rash was a papular erythema; the papules then became confluent and surrounded by erythematous areas. This subsided in about a week, and was followed by desquamation. In one case a portion of the eruption was urticarial, in another the papules were petechial, and in a third vesicles developed. He points out that there is a considerable similarity to the eruptions which occasionally occur in pyæmia and in ptomaine poisoning. Of the five cases, four ended fatally from uræmia.

[10] Brit. Jour. of Derm., May, 1899, p. 194. [11] Brit. Med. Jour. Epit., Jan., 1899; Munch. Med. Woch., Dec. 6, 1898. [12] Practitioner, May, 1899, p. 609. [13] Practitioner, May, 1899, p. 602; Glasgow Hosp. Rep., Vol. I., p. 53, 1898. [14] Brit. Jour. of Derm., May, 1899, p. 188. [15] Brit. Jour. of Derm., May, 1899, p. 193. [16] Brit. Jour. of Derm., June 11, 1899, pp. 193, 221. [17] Brit. Jour. of Derm., July, 1899, p. 276.

